# Supplementary material for: Stability and dynamics of a spectral graph model of brain oscillations
Source: Netw Neurosci. 2023 Jan 1;7(1):48–72. doi: 10.1162/netn_a_00263 (PMC10270709; doi:10.1162/netn_a_00263)
Supplement: Supplementary file 1 [file netn-7-1-48-s001.pdf]

Verma, P., Nagarajan, S. & Raj, A. (2023). Supporting information for "Stability and dynamics of a spectral graph model of brain oscillations." *Network Neuroscience*, *7*(1), 48–72.
https://doi.org/10.1162/netn_a_00263

Stability and dynamics of a spectral graph model of brain oscillations

Parul Verma Srikantan Nagarajan Ashish Raj

Department of Radiology and Biomedical Imaging
University of California San Francisco, USA

Supplementary Figures

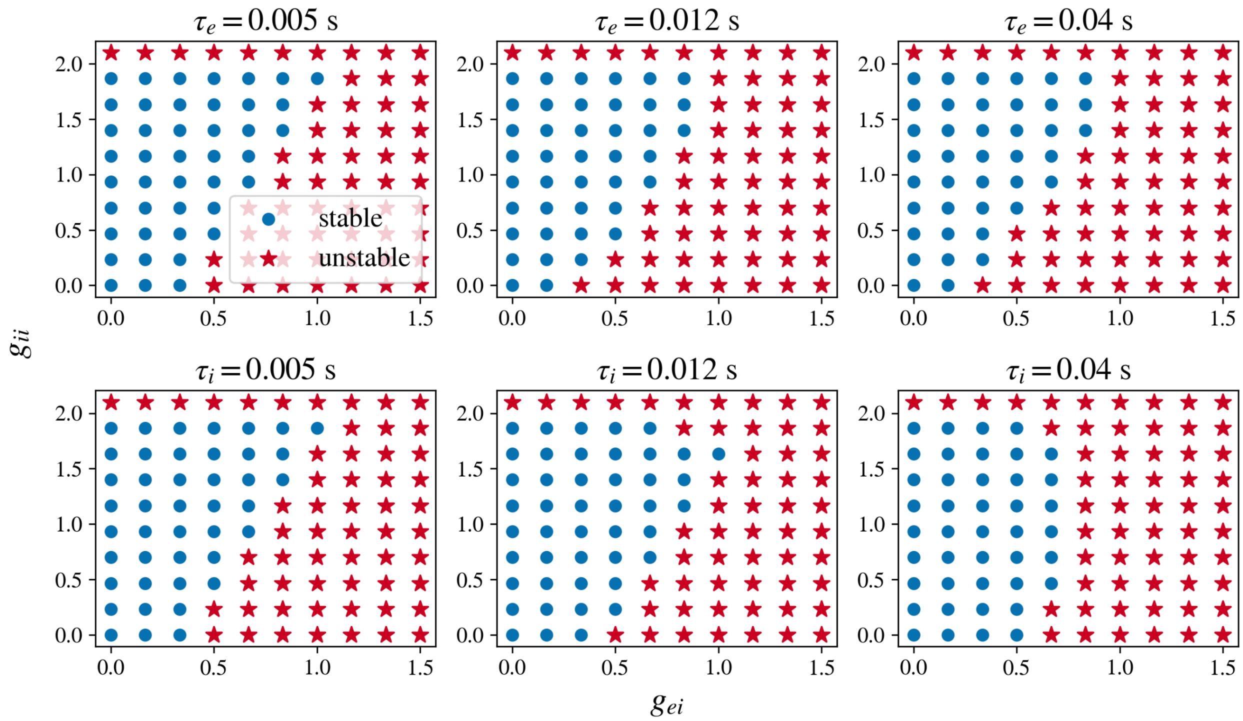

Figure S1: Stability regime for mesoscopic model. Stability regimes plotted for different values of τ_e and τ_i. When only τ_e is varied, τ_i is fixed at 0.003. When only τ_i is varied, τ_e is fixed at 0.012. Blue dots represent stable parameter combinations and red stars represent unstable ones.

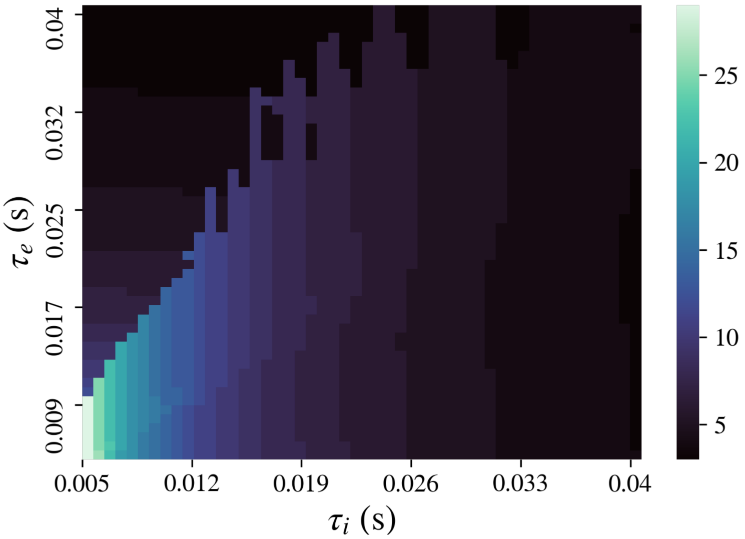

Figure S2: Frequency at which the primary peak is observed in the modeled mesoscopic frequency spectra upon varying τ_e and τ_i simultaneously. Peak in the gamma region can be obtained for very low values of τ_e and τ_i both. For most of the parameter values, a peak in the lower frequency range is observed. Here, $g_\mathrm{ii} = 1.5$ and $g_\mathrm{ei} = 0.25$ to ensure stability.

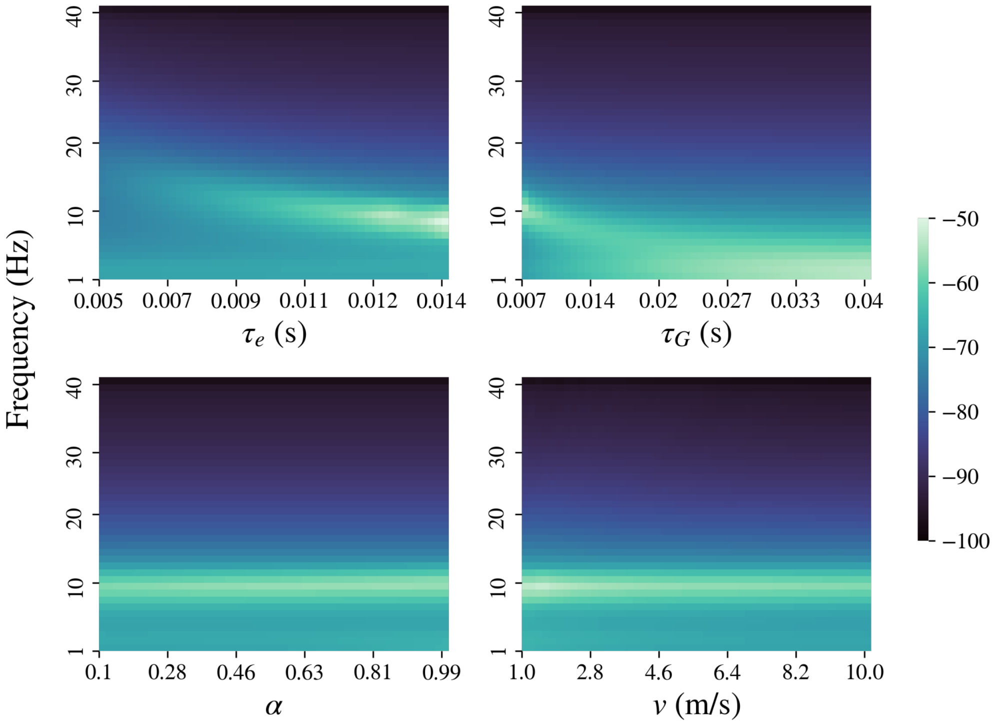

Figure S3: Macroscopic modeled frequency spectra (averaged over all regions) obtained by varying model parameters: **A:** τ_{e}, **B:** τ_{G}, **C:** α, and **D:** v. This spectra is obtained when the macroscopic model's input $x_{\mathrm{e}}(t) + x_{\mathrm{i}}(t)$ is replaced with $\exp(-t)$. The default parameters are $\alpha = 0.5$, $v = 5$ m/s, $\tau_{\mathrm{e}} = 0.012$, and $\tau_{\mathrm{G}} = 0.008$ s.

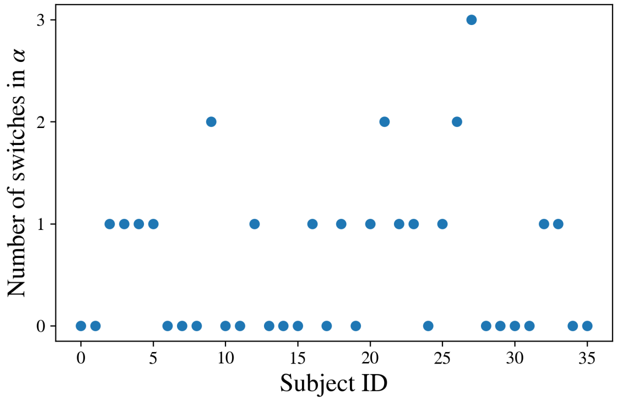

Figure S4: Number of switches in α. Here, the upper bound on α is 1. Switches were observed for 17 out of 36 subjects.

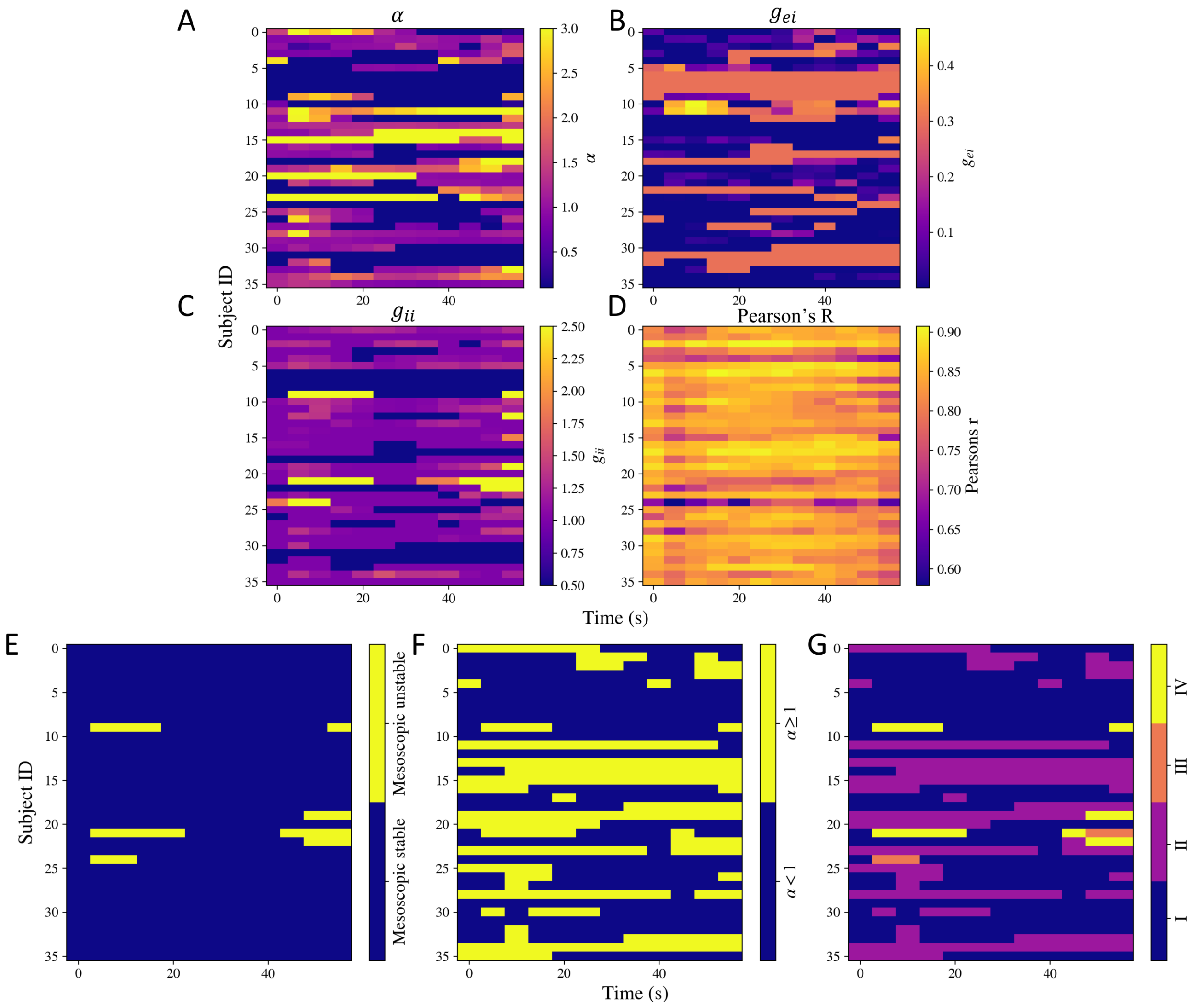

Figure S5: Estimated model parameters **A:** α, **B:** $g_{\rm ei}$, **C:** $g_{\rm ii}$, and **D:** the goodness of fit Pearson's r calculated at different time points for all the subjects, keeping $\alpha = 3$ as the upper bound. **E, F, G: Dynamic stability. E:** Stability of the mesoscopic model over time. **F:** Switches in α over time. **G:** Switches in different regimes of stability over time. The shade is based on 4 situations: I) both mesoscopic model is stable and $\alpha < 1$, II) mesoscopic model is stable but $\alpha \geq 1$, III) mesoscopic model is unstable but $\alpha < 1$, IV) mesoscopic model is unstable and $\alpha \geq 1$.

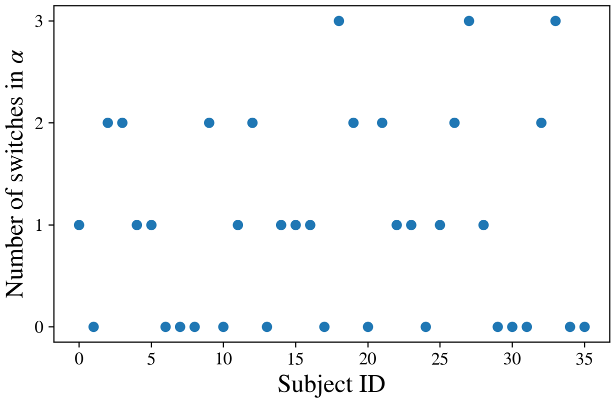

Figure S6: Number of switches in α, keeping the upper bound of α at 3. Switches were observed for 22 out of 36 subjects.

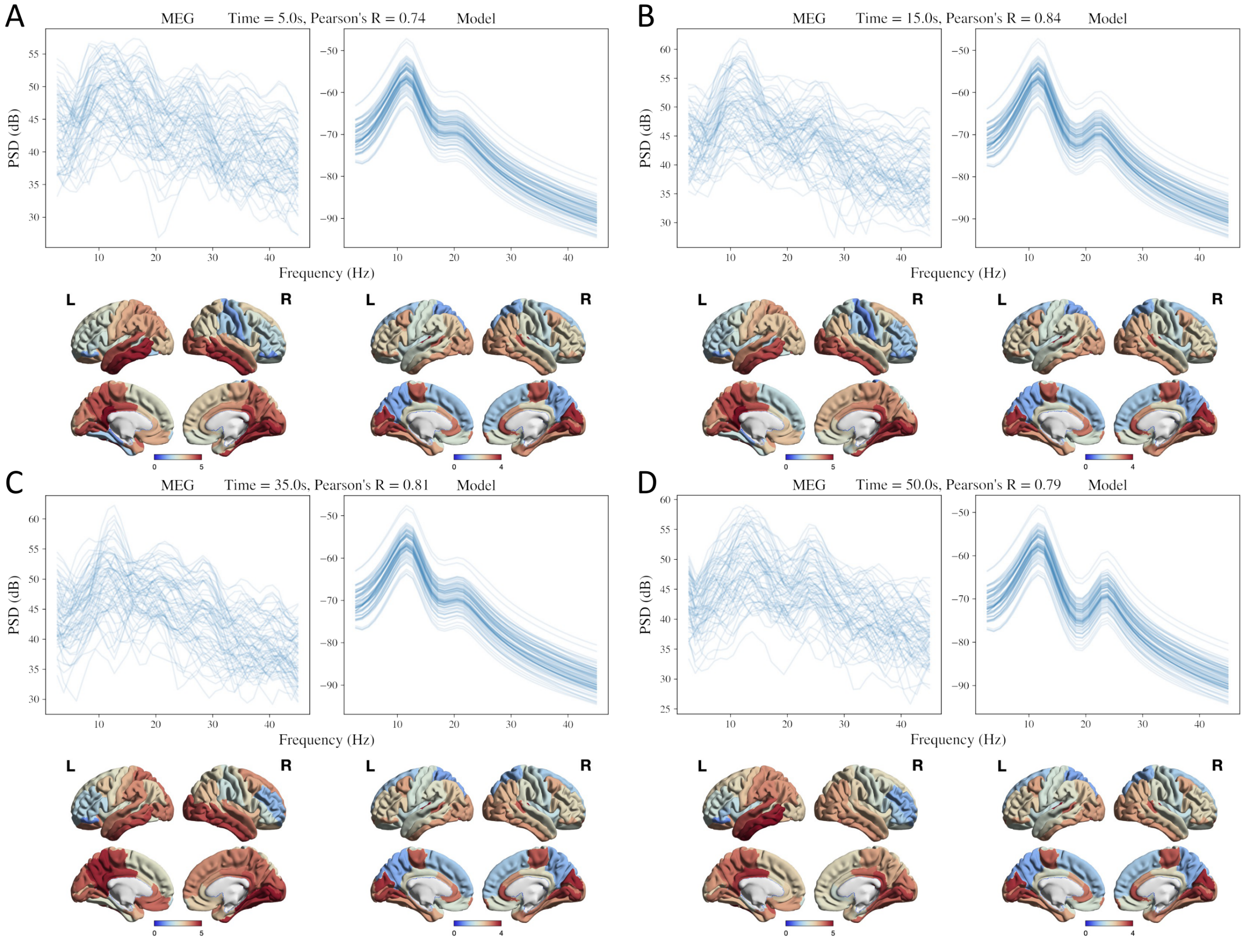

Figure S7: Empirical versus modeled spectra for a specific subject. The top subplots demonstrate the empirical and modeled frequency spectra at different time points. The bottom brain surface renderings demonstrate the empirical and modeled spatial distribution of alpha frequency band at the corresponding time points. Alpha frequency band spatial distribution is obtained by summing up the empirical and modeled frequency spectra between frequencies of 8-12 Hz.

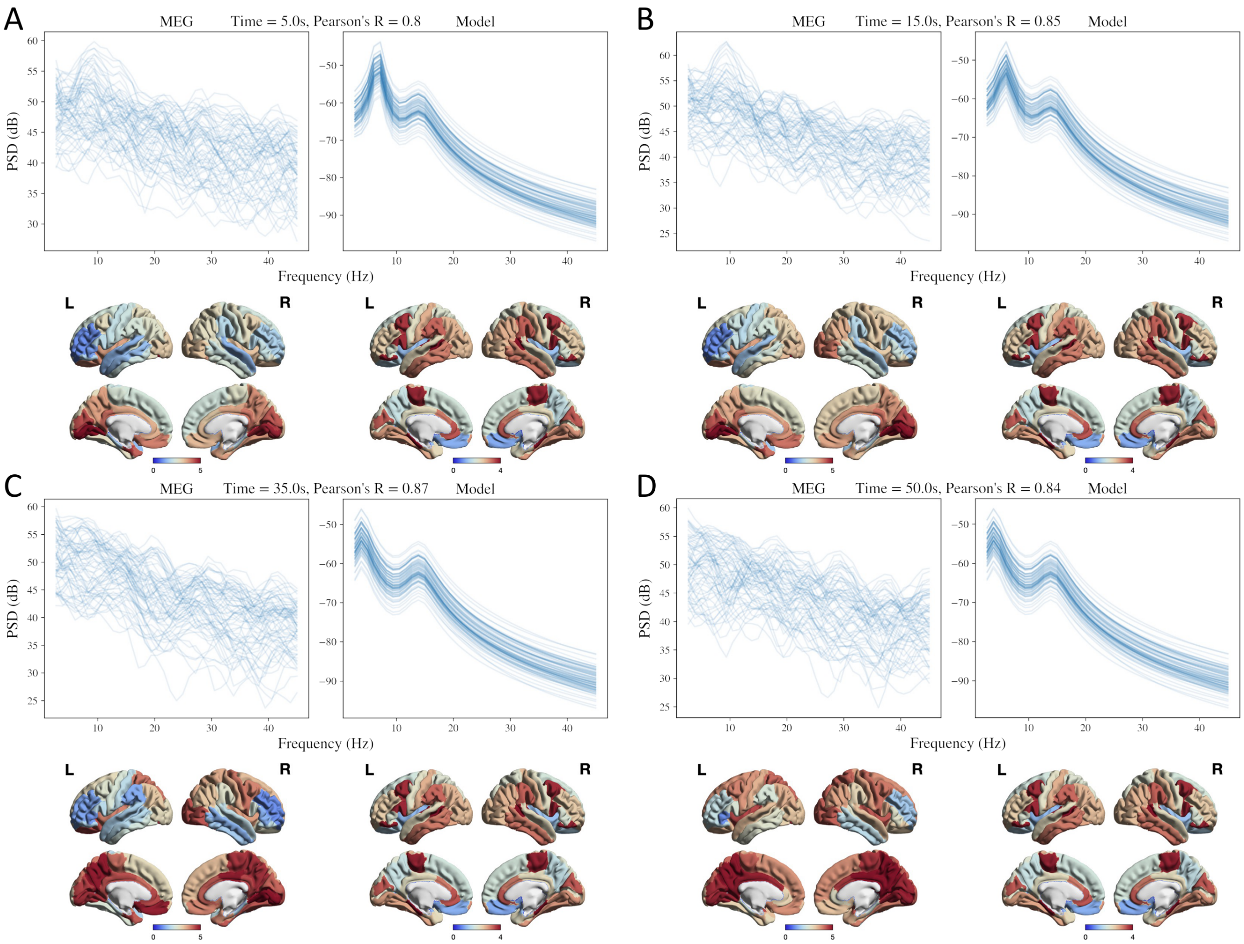

Figure S8: Empirical versus modeled spectra for a specific subject. The top subplots demonstrate the empirical and modeled frequency spectra at different time points. The bottom brain surface renderings demonstrate the empirical and modeled spatial distribution of alpha frequency band at the corresponding time points. Alpha frequency band spatial distribution is obtained by summing up the empirical and modeled frequency spectra between frequencies of 8-12 Hz.

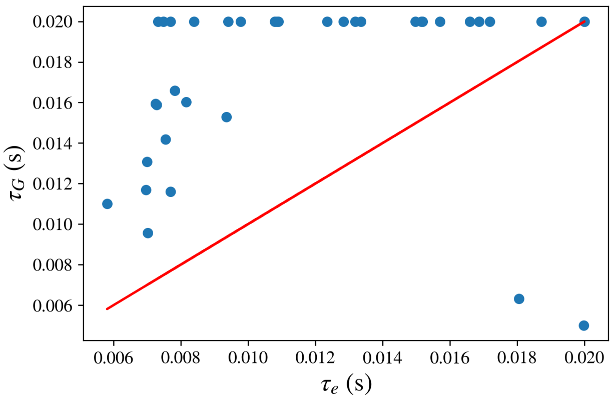

Figure S9: Estimated time constants τ_{G} versus τ_{e} for the static spectra of each of the subjects. The red line corresponds to the diagonal line and every point corresponds to a subject. For two subjects, τ_{G} was much lower, implying the system is unstable.
